# Lipid and brain volumetric measures in multiple sclerosis patients: findings from a large observational study

**DOI:** 10.1007/s13760-024-02676-w

**Published:** 2024-10-25

**Authors:** Balazs Lorincz, Michal Vrablik, Ramanathan Murali, Eva Kubala Havrdova, Dana Horakova, Jan Krasensky, Manuela Vaneckova, Tomas Uher

**Affiliations:** 1 Department of Neurology, Center of Clinical Neuroscience, First Faculty of Medicine, Charles University in Prague and General University Hospital, Prague, Czech Republic; 2 Third Department of Internal Medicine, Department of Endocrinology and Metabolism, First Faculty of Medicine, Charles University in Prague and General University Hospital, Prague, Czech Republic; 3 Department of Pharmaceutical Sciences and Neurology, University at Buffalo, State University of New York, Buffalo, NY USA; 4 Department of Radiology, First Faculty of Medicine, Charles University in Prague and General University Hospital, Prague, Czech Republic

**Keywords:** Lipid, Cholesterol, LDL, HDL, Brain atrophy, Lesion volume, MRI, Multiple sclerosis

## Abstract

**Objectives:**

This study aimed to investigate relationships between cholesterol profile, brain volumetric MRI, and clinical measures in a large observational cohort of multiple sclerosis (MS) patients.

**Materials and methods:**

We included 1.505 patients with 4.966 time points including complete lipid, clinical, and imaging data. The time among lipid, brain MRI and clinical measures was under 90 days. Cross-sectional statistical analysis at baseline was performed using an adjusted linear regression and analysis of longitudinal lipid and MRI measures data was performed using adjusted linear mixed models.

**Results:**

We found associations between higher high-density lipoprotein cholesterol (HDL-C) and lower brain parenchymal fraction (BPF) at cross-sectional analysis at baseline (B = −0.43, CI 95%: −0.73, −0.12, *p* = 0.005), as well as in longitudinal analysis over follow-up (B = −0.32 ± 0.072, χ^2^ = 36.6; p = < 0.001). Higher HDL-C was also associated with higher T2-lesion volume in longitudinal analysis (B = 0.11 ± 0.023; χ^2^ = 23.04; p = < 0.001). We observed a weak negative association between low-density lipoprotein cholesterol (LDL-C) levels and BPF at baseline (B = −0.26, CI 95%: −0.4, −0.11, p = < 0.001) as well as in longitudinal analysis (B = −0.06 ± 0.03, χ^2^ = 4.46; *p* = 0.03). T2-LV did not show an association with LDL-C. We did not find any association between lipid measures and disability. The effect of lipid levels on MRI measures and disability was minimal (Cohen f2 < 0.02).

**Conclusions:**

Our results contradict the previously described exclusively positive effect of HDL-C on brain atrophy in patients with MS. Higher LDL-C was weakly associated with higher brain atrophy but not with higher lesion burden.

## Introduction

Multiple sclerosis (MS) is a chronic, demyelinating, and neurodegenerative disease of the central nervous system (CNS). Several environmental and lifestyle factors have been suggested to affect the course of the disease and disability progression [1]. Cholesterol homeostasis in the CNS environment is highly regulated and not yet completely understood [2]. Similarly, to other autoimmune diseases (e.g., rheumatoid arthritis, systemic lupus erythematosus) [3, 4] a disrupted lipid profile is a common finding among MS patients. People with MS also have a higher prevalence of cardiovascular risk factors [5]. Several markers of lipid metabolism and cholesterol turnover have been associated with radiological disease activity, brain atrophy and faster disability accrual [6, 7].

Although the association between changes in lipid measures and the rate of brain volume loss and accumulation of lesion burden are reported in several studies [7–11], it is not clear whether these associations also hold for absolute lipid levels. This is of great importance to better understand the role of lipid metabolism in MS, because it is not clear whether modifications of lipid metabolism through medication or lifestyle changes have the potential to affect disease activity in MS. Thus, this study aimed to evaluate the association between lipid and brain volumetric MRI measures in a large heterogeneous population of patients with MS.

## Materials and methods

### Study design

We included 1.505 patients with 4.966 timepoints from the MS Centre in the General University Hospital in Prague with available lipid measures between 1/2000 and 12/2015. Inclusion criteria included a confirmed diagnosis of relapsing remitting (RRMS), primary or secondary progressive MS (PMS) based on McDonald 2017 criteria [12], availability of Expanded Disability Status Scale (EDSS), lipid measures and brain MRI data suitable for volumetric analysis. The time between the lipid, clinical and MRI measures did not exceed 90 days.

Clinical and demographic data in this study from our MS center were collected via the Czech Republic nationwide registry (ReMuS). The guarantor of expertise of ReMuS is the Section for Neuroimmunology and Liquorology of the Czech Neurological Society. Data in ReMuS are collected using the standardized software iMed. Before the release of the coded patient-level data to investigators, there was performed a multi-level data quality control process (please add reference here: Stastna D., et al., The Czech National MS Registry (ReMuS): data trends in multiple sclerosis patients whose first disease-modifying therapies were initiated from 2013 to 2021. Biomed Pap Med Fac Univ Palacky Olomouc Czech Repub, 2023). The study protocol was approved by the Medical Ethics Committee of General University Hospital in Prague (113/22 S-IV). The study was carried out in accordance with the Declaration of Helsinki, and all patients provided written informed consent.

## Imaging methods

MRI measures were obtained during regular follow-up of routine clinical practice at the Department of Radiology at the General University Hospital in Prague. A standardized protocol was performed using a single 1.5T scanner (Gyroscan; Philips Medical Systems, Best, The Netherlands). Axial brain acquisitions included fluid-attenuated inversion recovery (FLAIR) and three-dimensional (3D) T1-weighted images (WI). Volumetric measures of the brain parenchymal fraction (BPF) and T2-lesion volume (T2-LV) were obtained using the previously described in-house software ScanView. Comparison of accuracy of ScanView technique with commonly used volumetric software was published previously [13].

## Biological samples and measurements

Considering that fasting state has not been found to be superior to non-fasting state when evaluating a lipid profile [14] fasting or non-fasting blood draws were obtained in routine clinical practice. Diagnostic reagent kits (Cholesterol Liquicolor and HDL Liquicolor; Human Gesellschaft fűr Biochemica und Diagnostica mbH, Germany) were used to measure serum total cholesterol (TC), high-density lipoprotein cholesterol (HDL-C) and triglyceride (TAG). Low-density lipoprotein cholesterol (LDL-C) was obtained using the Friedewald equation (all patients had TAG < 4.2).

### Data analysis

We used the R programming language (version 4.2.0) for data analysis. The R Studio (version 2022.02.2 Build 485) interface was used. Demographic statistical analysis was performed with the TableOne package (version 0.13.2). The LME4 (version 1.1–29) package was used for linear mixed model analysis.

For baseline data, a linear regression model was fitted with baseline MRI measures (BPF, T2-LV) as the dependent variable, adjusted for age at baseline, disease duration at baseline, gender, EDSS at baseline, and disease-modifying therapy (DMT) status at baseline (no DMT, DMTs with low to moderate efficacy, DMTs with high efficacy). We grouped interferons, glatiramer acetate, teriflunomide and dimethyl fumarate as DMTs with low to moderate efficacy, other DMTs (natalizumab and fingolimod) were considered as high efficacy treatment.

For longitudinal data, a linear mixed effect model with a random intercept for the patient and a random slope for the operator variable (lipid measure) was used. The models were adjusted for age, disease duration, gender, EDSS, and DMT status (no DMT, DMTs with low to moderate efficacy, DMTs with high efficacy). To further elaborate on the results of longitudinal analysis we adjusted models also for disease phenotype treated as a categorical variable (RRMS or PMS). Mixed models with an interaction term were used to investigate additive effect of age, disease duration, EDSS and disease phenotype on association between lipid and imaging measures.

In the sensitivity analysis, we included only patients with LDL-C < 3.0 mmol/l to exclude potential effect of hypolipidemic drugs and outliers on our results.

Statistical significance was assessed by comparing the models to a null hypothesis using analysis of variance. We estimated effect size (Cohen f2) for the effect of lipid measures on the imaging metrics. Cohen f2 ≥ 0.02, f2 ≥ 0.15, and f2 ≥ 0.35 represent small, medium, and large effect sizes, respectively [15].

## Data availability

Anonymized data not published in this article will be made available upon reasonable request from a qualified investigator.

## Results

### Demographic and clinical characteristics of patients

We included 1.505 patients with 4.966 time points over time. The mean age at first MRI scans was 37.25 (SD ± 10.55) years, 70.3% were females, median EDSS at baseline was 2.0 (IQR: 1.0, 3.0), and mean disease duration at baseline was 9.05 (SD ± 8.00) years. Patients were using low to moderate efficacy DMT in 72.3% of timepoints, high efficacy DMT in 10.6% timepoints and during the 17.1% of timepoints, patients did not use any DMT. At baseline, the mean TC was 4.94 mmol/l, mean HDL-C was 1.56 mmol/l, mean LDL-C was 2.92 mmol/l. More details are provided in Table 1.

**Table 1:** Demographic, clinical, biochemical and MRI characteristics of patients at baseline

Variable	Overall
Number of patients included	1505
Number of total timepoints	4966
MS subtypes at baseline (%)	
RRMS	1238 (82)
PMS	267 (18)
Total cholesterol (mean (SD)) (mmol/l)	4.94 (1.01)
TAG (mean (SD)) (mmol/l)	1.37 (0.88)
HDL-C (mean (SD)) (mmol/l)	1.56 (0.41)
LDL-C (mean (SD)) (mmol/l)	2.92 (0.80)
Gender = Male (%) (mmol/l)	446 (29.7)
EDSS (median (IQR))	2.0 (1.0,3.0)
T2 lesion volume (median (IQR)) (mm3)	1.52 (4.85)
Brain parenchymal fraction (mean (SD)) (%)	85.58 (2.30)
Age at MRI scans (mean (SD)) (years)	37.25 (10.55)
Disease duration at MRI scans (mean (SD)) (years)	9.05 (8.00)
DMT usage by groups at baseline (%)	
No DMT	398 (26.4)
DMTs with low to moderate efficacy	918 (61.0)
DMTs with high efficacy	189 (12.6)

RRMS = relapsing remitting multiple sclerosis, PMS = (primary or secondary) progressive multiple sclerosis TAG = triglycerid, HDL-C = High-density lipoprotein cholesterol, LDL-C = Low-density lipoprotein cholesterol, EDSS = Expanded disability status scale, DMT = Disease-modifying treatment

We conducted a check for hypolipidemics use in 300 random patients with LDL-C levels higher than 3.0mmol/l revealing that only a negligible proportion of patients (< 2%) were using lipid-level stabilizing medication.

## Associations among lipid, MRI, and clinical measures at baseline

We did not find an association between TC and MRI measures. We found an association between higher HDL-C and lower BPF (B = −0.43; CI 95% = −0.73, −0.12; *p* = 0.005). This association remained present, even when HDL-C/TC ratio (B = −2.66; CI 95% = −4.2, −1.05; *p* = 0.001) was used as a predictor instead of HDL-C. Lower LDL-C was also associated with higher BPF (B = −0.26; CI 95%: −0.4, −0.11; p = < 0.001).

T2-lesion volume (T2LV) was not associated with HDL-C (B = −0.19; CI 95% = −1.4, 1.03; *p* = 0.77) or LDL-C levels (B = −0.3; CI 95% = −0.9, 0.31; *p* = 0.33).

Baseline HDL-C, HDL-C/TC, and LDL-C measures were not associated with EDSS.

However, BPF (B = −0.02; CI 95% = −0.03, −0.01; p = < 0.001), as well as T2-LV (B = 0.009; CI 95% = 0.006, 0.01; p = < 0.001), were associated with EDSS.

**Fig. 1:**
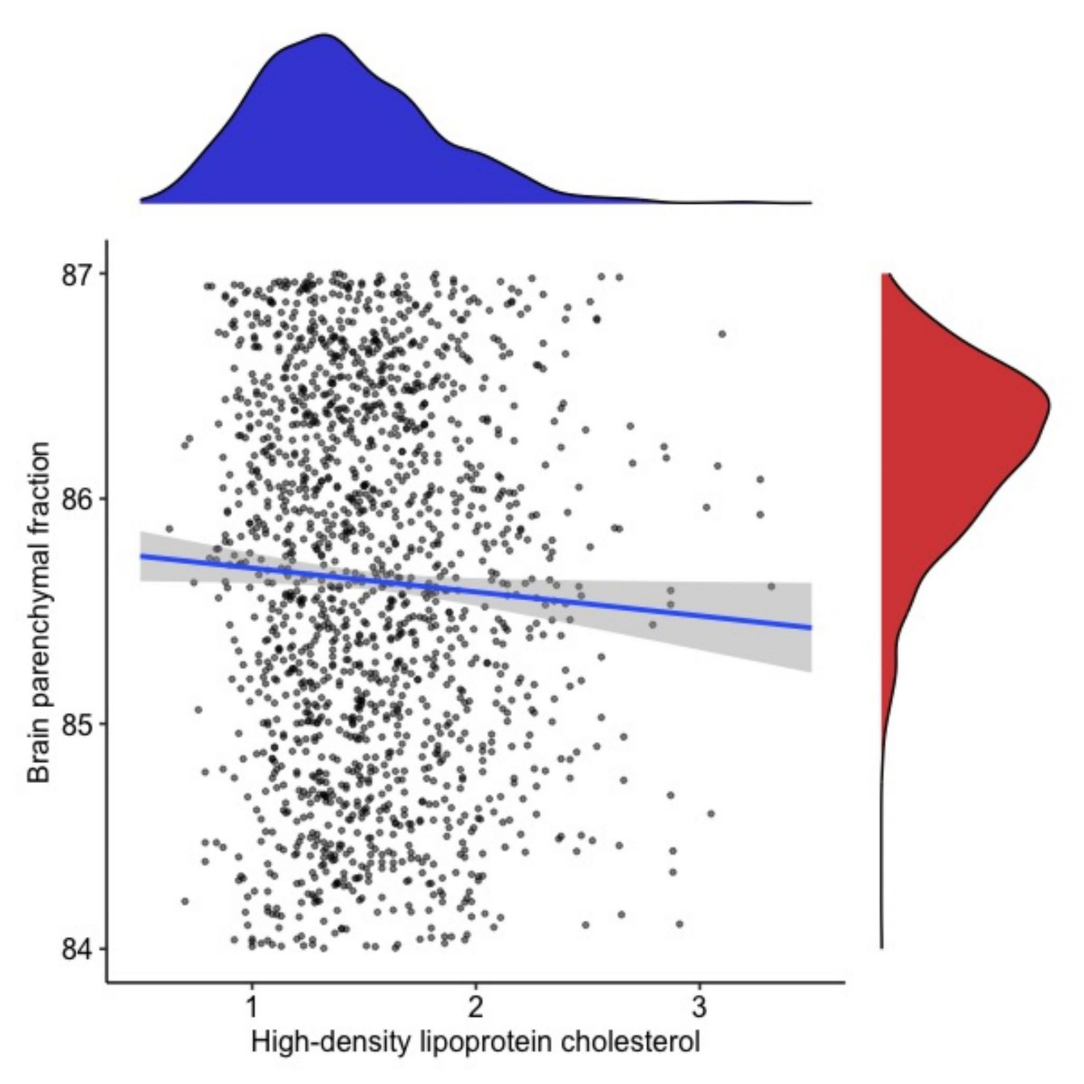
The association between high-density lipoprotein cholesterol (HDL-C) and brain parenchymal fraction (BPF) and data distribution

**Fig. 2:**
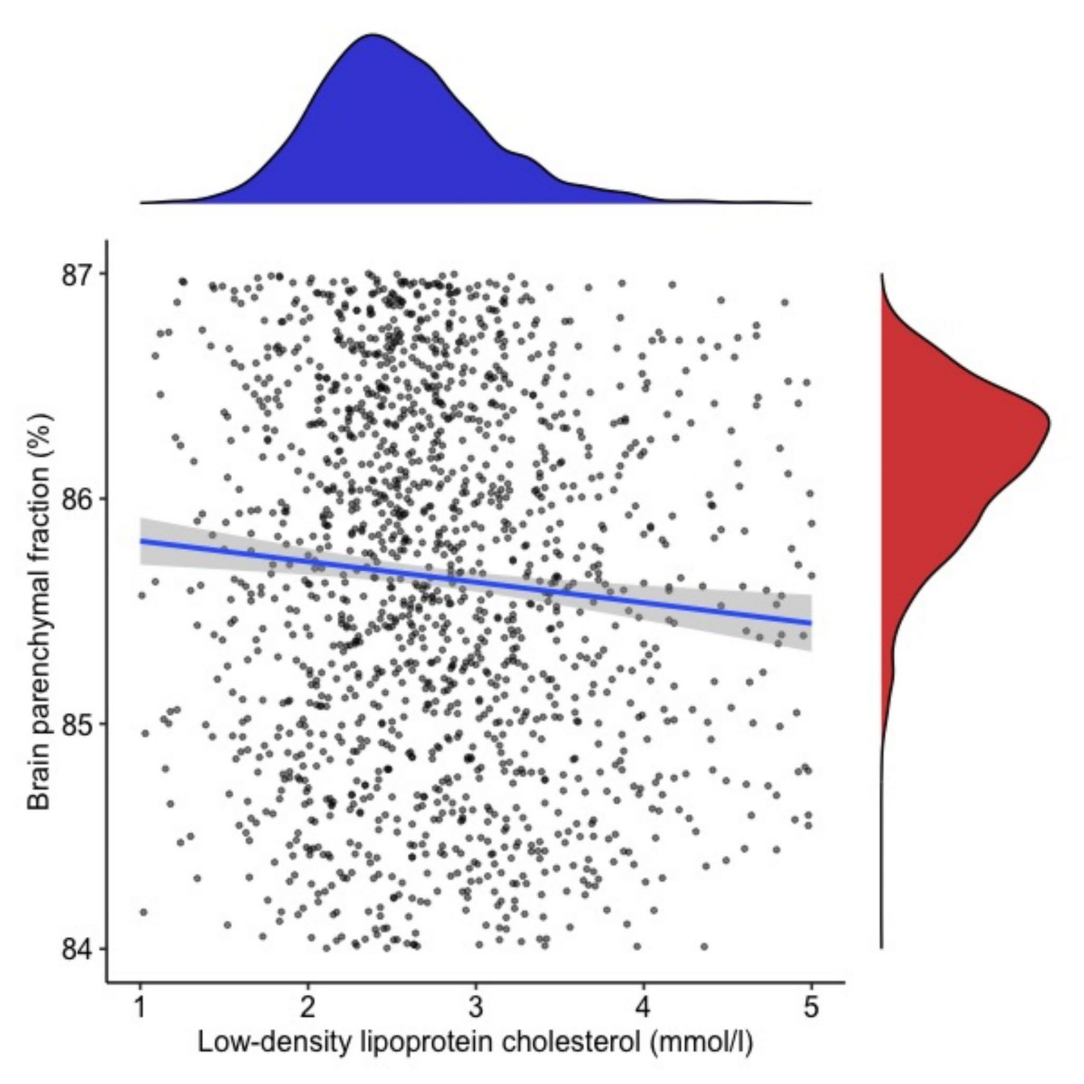
The association between low-density lipoprotein cholesterol (LDL-C) and brain parenchymal fraction (BPF) and data distribution

The effect of lipid profiles on MRI measures and disability was minimal (all Cohen´s f2 < 0.02) (Table 2, Figs. 1, 2). If analyzing only patients with LDL-C levels < 3.0 mmol/l, we observed very similar results (data not shown).

**Table 2:** Summary of baseline and longitudinal results

	Baseline		Follow-up	
	BPF	T2-LV	BPF	T2-LV
TC	B = − 0.025 95%CI = −0.13, 0.084 *p* = 0.65	B = 0.003 95% CI = −0.05, 0.05 *p* = 0.89	B = −0.08 *p* = 0.1	B = −0.11 *p* = 0.25
LDL-C	**B= −0.26** **95%CI = −0.40**,** −0.11** **p = < 0.001**	B = −0.32 95% CI = −0.9, 0.31 *P* = 0.33	**B = −0.06 ± 0.03** **χ**^**2**^ **= 4.46** *p* = 0.03	B = −0.26 *p* = 0.31
HDL-C	**B= −0.43** **95%CI = −0.73**,** −0.12** *p* = 0.005	B = 0.19 95% CI = −0.21, 0.03 *p* = 0.77	**B = −0.32 ± 0.072** **χ**^**2**^ **= 36.6;** **p = < 0.001**	**B = 0.11 ± 0.023** **χ**^**2**^ **= 23.04** **p = < 0.001**
Tg	B = −0.11 95% CI = −0.24, 0.01 *p* = 0.08	B = −0.02 95% CI = −0.07, 0.03 *P* = 0.39	B = −0.13 *p* = 0.15	B = −0.13 *p* = 0.24

TC = Total Cholesterol, Tg = triglycerid, HDL-C = High-density lipoprotein cholesterol, LDL-C = Low-density lipoprotein cholesterol, BPF = Brain Parenchymal Fraction, T2-LV = T2 lesion volume

### Association among lipid, MRI, and clinical measures over follow-up

Changes in TC did not affect BPF. Increase in HDL-C was associated with decreased BPF (B = −0.32 ± 0.072; χ^2^ = 36.6; p = < 0.001) and increased T2-LV (B = 0.11 ± 0.023; χ^2^ = 23.04; p = < 0.001). In other words, increase of HDL-C by 1 mmol/l decreased BPF by 0.32%, and increased T2LV by 0.11 mm^3^. Increased LDL-C was associated with slightly lower BPF (B = −0.06 ± 0.03; χ^2^ = 4.46; *p* = 0.03). When adjusting only for age and gender the results did not change significantly.

When introducing disease phenotype (RRMS or progressive MS) as an interaction term to HDL-C or LDL-C we did not observe significant results, meaning that disease phenotype has no effect on the previously described associations between lipid and MRI measures. The association between BPF and HDL-C was stronger with increasing disease duration (interaction between HDL-C and disease duration, B = 0.02; *p* = 0.05). Similarly, the association between T2-LV and HDL-C was getting stronger by longer disease duration (interaction between HDL-C and disease duration, B = 0.08; *p* = 0.05). However, the effect of disease duration on the investigated correlations was minimal. In addition, we did not find any effect of age and EDSS on the correlations between lipid and imaging measures.

We confirmed negative association between BPF and EDSS in the longitudinal analysis (B = −0.311 ± 0.084; χ^2^ = 13.06; p = < 0.001). The lack of association between lipid measures and EDSS overtime was in line with cross-sectional analysis.

The effect of lipid profiles on MRI measures and disability was minimal (all Cohen´s f2 < 0.02) (Table 2). If analyzed only patients with LDL-C levels < 3.0 mmol/l, we observed very similar results (data not shown).

## Discussion

HDL-C promotes the efflux of cholesterol from cells and has antioxidant and anti-inflammatory effects [16]. Higher baseline levels of HDL-C, as well as Apolipoprotein A1 (ApoA1), which is the characteristic apolipoprotein of HDL, were previously shown to be associated with a lower number and volume of contrast-enhancing lesions [17], with better blood-brain barrier (BBB) function [18], and with lower levels of neurofilaments [19]. Relative increase in HDL-C was also associated with slower cortical and white matter volume loss [8, 11]. Based on these results, a positive effect of HDL-C on the progression of MS was suggested.

However, previous studies focused primarily on the correlations between relative changes in lipid and MR measures over time, rather than absolute values of lipid and imaging measures. In this study, we investigated absolute lipid and imaging measures and found that the higher absolute HDL-C values were not associated with decreased brain atrophy, as would be expected based on the previous research. Conversely, HDL-C was surprisingly associated with a slightly lower normalized brain volume in both cross-sectional and longitudinal analyses. This negative correlation remained significant even when HDL-C was substituted with the HDL-C/TC ratio which accounts for contribution of HDL-C to TC levels. It is needed to be emphasized that our sample included a large sample of patients. Therefore, the statistical analysis was overpowered and able to detect minuscule effects of lipid levels on MRI measures. In this context, we cannot exclude that negative association between high HDL-C and brain atrophy found in our study is just a random finding. Therefore, this observation should be interpreted with great caution. We acknowledge that numerous external factors can impact HDL-C levels, leading to its increased or decreased level. In our specific context, it is important to note that the HDL-C decreases during systemic inflammation [20] or using of interferons [9], but increases following alcohol intake [21], using of S1P modulators or dimethyl fumarate [22]. However, MS is not associated with systemic inflammation [23], all patients in our study had normal CRP levels (except of 20 (< 1%) measurements) and normal hepatic enzymes levels suggesting potential regular alcohol misuse in only marginal proportion of patients. Only very few patients were treated with either S1P modulators (*n* = 348 of all timepoints, *n* = 125) or dimethyl fumarate (*n* = 7 of all timepoints, *n* = 7 of all patients) and statistical analyses were adjusted for DMT making an effect of treatment on our results minimal. In addition, in sensitivity analysis including only patients with normal LDL-C levels, we observed very similar results and only negligible number of patients with high LDL-C levels were using hypolipidemic drugs.

We also found a negative association between LDL-C and BPF in the cross-sectional and longitudinal analysis. Although, this observation confirms findings of the previous research the associations found in our and recent study are weak [10, 24].

Taken together our findings undermine the previously suggested exclusive protective properties of higher HDL-C levels in MS.

In this context, it should be noted that other autoimmune disorders (e.g., rheumatoid arthritis, systemic lupus erythematosus) showed altered HDL functioning with pro-inflammatory properties [25, 26]. Similarly, a recent study in MS patients showed an increased proportion of dysfunctional HDL particles [27]. The pathological behavior of some HDL species was attributed to the modification of the anti-inflammatory and antioxidative characteristics of HDL under systemic inflammatory conditions [28]. These changes were most prominent in small HDL particles [29]. One of the suggested mechanisms of the conversion of small HDL particles into their pro-inflammatory form is lipoprotein peroxidation [30]. This process causes oxidative stress which accelerates demyelination and neurodegeneration [31]. In this context it is important to note that in mice models physical exercise decreased all of the above explained adverse effects [32]. Several studies, although on a small number of patients, showed higher small-HDL concentrations in MS patients compared to age-matched healthy controls [24, 33].

Moreover, recently several studies have shown the possible negative effects of increased HDL-C on CNS pathology. For example, post-hoc analysis of the Aspirin in Reducing Events in the Elderly (ASPREE) trial [34] performed on around 19.000 elderly participants showed that very high levels of HDL-C is associated with 27% higher risk of developing all-cause dementia over follow-up [35]. The explanation, why high HDL-C might be associated with an increased risk of dementia is unknown. The authors of this study emphasized that plasma HDL-C levels might not reflect functional aspects of lipid transport and that very high levels HDL-C may have dysfunctional properties [36]. In addition, it was suggested that it could be possible that increased risk of dementia and very high HDL are both consequences of a separate and unrelated pathology [35]. Based on these results, we speculate whether increased levels of dysfunctional small HDL particles might be associated with proinflammatory conditions leading to accelerated brain atrophy in MS. HDL-C levels reflect the sum of heterogeneous lipid particles with either pro or anti-inflammatory properties and future research needs to investigate whether different distributions of HDL subfractions in MS patients may explain our unexpected findings.

We are aware of the limitations of this study mainly being its retrospective character and lack of healthy control group. Also, we only have available basic lipid profile data and lack apolipoprotein and other more detailed lipidomic measures. Available comorbidity and comprehensive medication data would also be an important factor to consider for future research. Also, obtaining more comprehensive MRI volumetric data, which includes regional brain volumes, could offer deeper insights into the association between lipid metabolism and neurodegenerative processes in our patients. Finally, epidemiological studies investigating whether individuals with MS exhibit alterations in lipid levels compared to healthy controls require further exploration. All these limitations and questions should be addressed in the future research.

Although the findings of this study are of relatively small clinical relevance, they may add new information on the role of lipid metabolism in pathogenesis of MS and serve as a base for further HDL function and proteomic studies.
